# An unusual and malignant intussusception in a child

**DOI:** 10.1186/s13052-016-0283-2

**Published:** 2016-08-01

**Authors:** Gabriella D’Angelo, Lucia Marseglia, Marta Manti, Giovanni Stroscio, Daniela Impollonia, Salvatore Arena, Pietro Impellizzeri, Carmelo Salpietro, Carmelo Romeo, Eloisa Gitto

**Affiliations:** 1 Neonatal and Pediatric Intensive Care Unit, Department of Pediatrics, University of Messina, Messina, Italy; 2 Unit of Paediatric Genetics and Immunology, Department of Paediatrics, University of Messina, Messina, Italy; 3 Unit of Paediatric Surgery, Department of Paediatrics, University of Messina, Messina, Italy; 4 Neonatal Intensive Care Unit, Department of Pediatrics, University of Messina, Messina, Italy; 5 Department of Pediatrics, University of Messina, Via Consolare Valeria, 1, 98125 Messina, Italy

**Keywords:** Malignant tumour, Small intestine, Bowel obstruction, Surgery, Child

## Abstract

Intussusception is a common cause of bowel obstruction in the pediatric population. Malignant lesions account for up to 30 % of all cases of intussusception in the small intestine. We herein report an interesting case of ileo-colic intussusception caused by diffuse large B-cell lymphoma, in a child. The patient underwent laparoscopic right hemicolectomy. Pathologic evaluation revealed a diffuse large B-cell lymphoma.

In cases of intussusception, especially in the older age group of children, a high index of suspicion for malignant lymphoma of the bowel should be observed.

## Dear editor

Intussusception, defined as the telescoping of a segment of the gastrointestinal tract into an adjacent one, is often seen in paediatric age [1]. Gastrointestinal tumours, especially if they occur in the large bowel, cause intestinal intussusception in 63–68 % of cases [2]. Intussusception caused by diffuse large B-cell lymphoma (DLBCL), as a cause of acute abdomen, is rare, and, often, intussusception represents the first clinical sign, leading, potentially, to disease detection at an early stage [3].

**Fig. 1:**
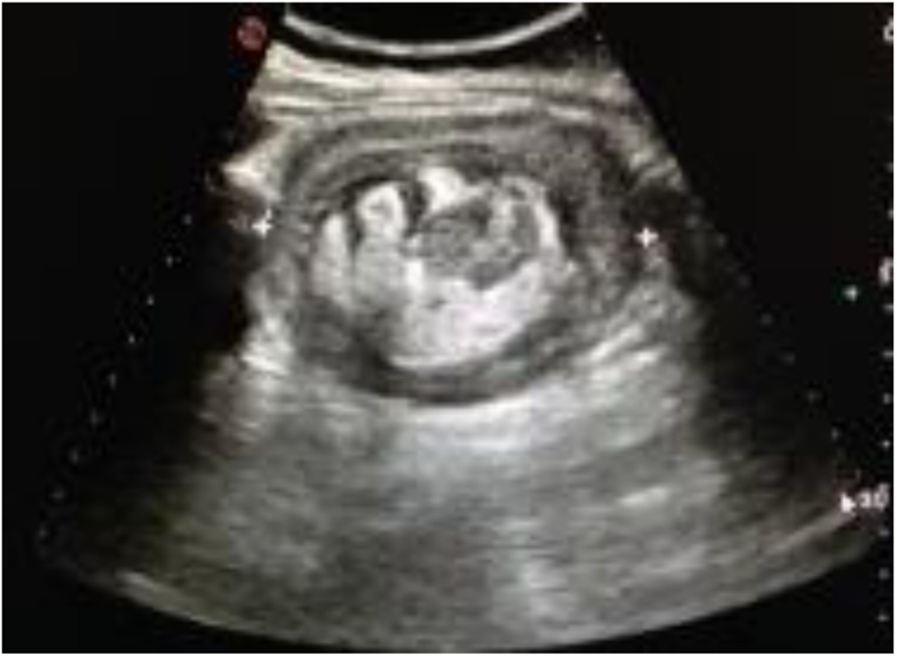
Abdominal ultrasound noted an intussusception, giving the typical “target” sign

**Fig. 2:**
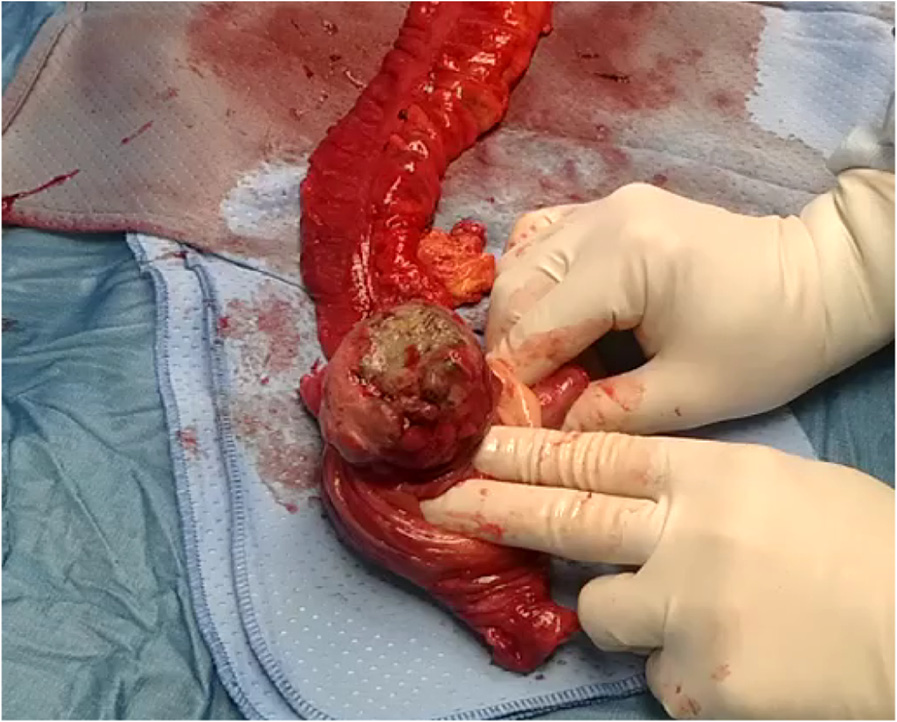
Resected specimen showing the large B-cell lymphoma

We herein present a rare case of an ileo-colic intussusception caused by DLBCL in a pediatric patient who initially underwent surgical treatment and, subsequently, adjuvant chemotherapy. An 8-year-old boy presented with previous medical history of abdominal pain and nausea with vomiting following meals. Symptoms progressively worsened to severe anorexia, weight loss, constipation, and bilious vomiting, on the day of admission. Fever, chills, bleeding per rectum, and previous abdominal surgeries were absent. Laboratory tests only showed increased C-reactive protein (2.5 mg/L, normal range 0–0.5 mg/L). Abdominal X-ray revealed no specific bowel gas pattern, but with gaseous distension of several small bowel loops. Focused sonography of the right lower quadrant reported a “target sign” or “doughnut”, pseudokidney/sandwich appearance [Fig. 1], suggesting bowel intussusception. Abdominal computed tomography (CT) revealed a typical target-appearing lesion, extending for approximately 16 cm of the terminal ileum and ascending colon into the transverse colon. Liver metastasis was also described. Emergency laparotomy confirmed a distal ileo-colic intussusception. Laparotomy was performed and an ileo-colic intussusception noted with a 4 cm × 4 cm intraluminal growth in the lumen, a large and hard mesenteric mass. Enlarged mesenteric lymph-nodes were also detected [Fig. 2]. The involved segment presented edema and initial necrosis with loss of the classic three-layer intestinal mucosa. It was resected with 5 cm margins, and end to end anastomosis was performed on the healthy small bowel. In the follow-up period, the patient underwent contrast enhanced CT scan of the thorax, abdomen and pelvis to look for synchronous lesions detected on admission (hepatic lesions). The child was thereafter referred to the Medical Oncology and Radiation Oncology Department where he underwent adjuvant chemotherapy and is under fortnightly follow-ups. The leading point of the invagination was a DLBCL measuring 4x4 cm, consisting of centroblastic/polymorphic cells of intermediate-large size; CD20+, CD10+, bcl2-, bcl6+, CD5-, D1, CKAE1-AE3, AML-. MIB > 70 %. All nodes showed reactive hyperplastic follicle-sinus histiocytosis and lymphostasis.

Primary malignant tumours of the small intestine are very rare, accounting for less than 2 % of all gastrointestinal malignancies [2]. Lymphoma constitutes 15–20 % of all small intestine neoplasms [4]. In particular, DLBCL, the most common form of non-Hodgkin lymphoma (NHL), is a heterogeneous entity rarely causing acute obstructive symptoms and intussusceptions [5]. Intestinal involvement of NHL has been correlated to increased frequency of abdominal symptoms resulting in an earlier diagnosis. It was found that the invasion depth of the tumour is significantly associated with patient survival [6]. DLBCL-patients initially undergo chemotherapy rather than surgery. Therefore, the relationship between surgery treatment and prognosis has not previously been reported. The importance of resection of the bowel containing even the smallest of lesions, along with removal of regional lymph nodes, is further stressed [7]. Moreover, a multicenter study found that primary surgical resection was associated with a favourable prognosis in cases of intestinal DLBCL, encouraging surgical resection as primary treatment [8]. Conversely, an increased risk of gastric adenocarcinoma after treatment of primary gastric lymphoma has been also reported, especially of diffuse large B-cell lymphoma [9]. Finally, there is no consensus on the optimal treatment against primary gastrointestinal DLBC [5, 6].

In conclusion, we describe a pediatric case of large B-cell lymphoma causing an ileo-colic intussusception. In cases of intussusception, especially in the older age group of children, a high index of suspicion for malignant lymphoma of the bowel, including DLBCL should be observed.

## Abbreviations

CT, computed tomography; DLBCL, diffuse large B-cell lymphoma; NHL, non-hodgkin lymphoma
